# Acute Effect of Gamma Irradiation on Gastric Acid Secretion and Gastric Mucosal Integrity in the Rat

**DOI:** 10.1100/tsw.2005.25

**Published:** 2005-03-18

**Authors:** Omar M. E. Abdel Salam, Ihsan Hadajat, Ayman Ragab Bayomy, Siham El-Shinawy, Mahmoud S. Arbid

**Affiliations:** ^1^ Department of Pharmacology, National Research Centre, Dokki, Cairo, Egypt; ^2^ Department of Biochemistry, National Centre for Radiation Research and Technology, Cairo, Egypt

**Keywords:** whole-body gamma irradiation, gastric acid secretion, rats

## Abstract

The effect of 3- or 6-Gray (Gy) whole-body gamma irradiation on basal and stimulated gastric acid secretion was studied in pylorus-ligated rats. Different groups of rats were irradiated with a single 3- or 6-Gy fraction and examined 7 days after irradiation. Exposure to 3-Gy fraction led to marked increase in basal (nonstimulated) gastric acid output in the 4-h pylorus-ligated rat (47.5% compared with unirradiated controls). After exposure to 6 Gy, only 18.2% increase in gastric acid output was noted compared with unirradiated controls. Under pentagastrin or histamine stimulation, gastric acid secretion in those irradiated with 3- or 6-Gy fraction was markedly reduced compared to that of unirradiated controls. Exposure to 3- or 6-Gy gamma irradiation intensified the degree of gastric mucosal injury evoked by indomethacin or 50% ethanol in a dose-dependent manner. It is concluded that in the pylorus-ligated rat model, lower doses of gamma irradiation increase basal gastric acid secretion and impair the gastric mucosal barrier with marked increase in its permeability to H^+^ following stimulation of acid secretion or exposure to barrier breakers. Exposure to irradiation is likely to result in failure of the parietal cell to respond to direct stimulation with histamine or pentagastrin.

## INTRODUCTION

Very little information is available regarding the effect of ionizing irradiation on gastric acid secretion and the gastric mucosa. Exposure to ionizing irradiation can have profound effects on gastric function. In rats, acute erosive and ulcerative gastritis and atonic dilatation of the stomach followed localized 300-kV X-ray irradiation (28.5 Gy) of the stomach[1,2]. In man, therapeutic pelvic irradiation is associated with widespread, persistent changes in gastrointestinal function[3]. Studies in rats and guinea pigs indicated that exposure to irradiation could suppress gastric acid secretion[4,5,6]. More recently, however, Lehy et al.[7] reported an increase in gastric acid output and plasma gastrin levels in rats exposed to 2- or 6-Gy whole-body gamma radiation. In the present study, the effect of whole-body gamma irradiation with 3 or 6 Gy on basal and histamine- or pentagastrin-stimulated gastric acid secretion was examined. We also assessed the ability of the gastric mucosa of rats irradiated with 3- or 6-Gy gamma radiation to withstand noxious chemical challenge following 50% ethanol or indomethacin under such conditions.

## MATERIALS AND METHODS

### Animals

Sprague-Dawley strain rats, 120–130 g of body weight, were used throughout the study and housed under standardized conditions for light and temperature. Rats were randomly divided into different groups (n = 6–7 per group) and were irradiated with a single 3- or 6-Gy fraction for 15–20 min and examined for gastric acid secretion or gastric ulcer 7 days later. Irradiation was performed through the use of $^{137}Cs$ gamma rays from the gamma cell-40 belonging to the National Centre for Radiation Research and Technology (NCRRT). The dose rate was 1 Gy/1.5 min at the time of the experiment. Rats were fasted for 18 h prior to experiments, but allowed water *ad libitum*, and kept in cages with wide meshed floors to prevent coprophagy.

### Study Design

#### *Gastric Acid Secretion Studies*

The effects of whole-body irradiation on gastric acid secretion were studied in pylorus-ligated rats and compared to that in unirradiated rats. Pylorus ligation was done according to Shay et al.[8]. Briefly, rats were lightly anesthetized with ether, a midline laparatomy was done to expose the pylorus, and a silk ligature was tied around the pylorus; care being care taken not to interfere with the blood supply to the stomach and duodenum. The abdominal wall was then closed in layers with silk sutures. Rats were administered 2 ml of physiological saline intragastrically (i.g.) through a soft orogastric tube. Rats were injected subcutaneously (s.c.) with 5 ml saline to correct for possible dehydration during the experiment.

The effect of gamma irradiation on gastric acid secretion was studied under basal (unstimulated) conditions and after stimulation of gastric acid secretion with pentagastrin (150 μg/kg) or histamine (1 mg/kg). The stimulants of gastric secretion were injected s.c., immediately after pylorus ligation. Rats were killed 4 h after pylorus ligation, the esophagus was then ligated, stomachs excised, and gastric juice carefully collected in graduated tubes after removal of the esophageal ligature. The volume of gastric secretion was noted and gastric acid output determined by automatic titration to pH 7.0 with 0.01*N* NaOH and $H^+$ output expressed as μEq/4 h/100 g.

#### *Gastric Ulcerogenic Studies*

The effects of indomethacin or ethanol on the gastric mucosa of rats irradiated with 3 or 6 Gy were studied and compared to that in control, unirradiated rats. Different groups of rats (n = 6–7 per group) were irradiated with a single 3- or 6-Gy fraction and examined 7 days after irradiation. Pylorus ligation was performed as described above, then gastric mucosal damage was evoked in pylorus-ligated rats by the administration of indomethacin (20 mg/kg s.c.) together with i.g. administration of 1 ml of physiological saline or by i.g. ethanol of 1 ml of 50% ethanol. Rats were injected with 5 ml saline s.c. to correct for possible dehydration during the experiment. Rats were killed 4 h later.

### Assessment of Gastric Mucosal Injury

Stomachs were opened along the greater curvature, rinsed with saline, extended on a plastic board, and examined for mucosal lesions. The number and severity of mucosal lesions were noted and lesions were scaled as follows: petechial lesions = 1, lesions less than 1 mm = 2, lesions between 1 and 2 mm = 3, lesions between 2 and 4 mm = 4, and lesions more than 4 mm = 5. A total lesion score for each animal is calculated as the total number of lesions multiplied by the respective severity scores. Results are expressed as the severity of lesions/rat[9].

### Histology

For histologic assessment, the stomachs were pinned flat on cardboard and immersed in 10% formalin solution and later embedded in paraffin. From the paraffin-embedded tissue blocks, hematoxylin and eosin–stained sections were coded. Sections were evaluated qualitatively under light microscopy.

### Determination of Gastric Mucus Content

Adherent gastric mucus was determined by the alcian blue recovery technique according to the method of Corne et al.[10]. Gastric mucus was expressed as μg/g wet tissue.

### Determination of Gastric Acid Secretion

In ulcer studies, after rats have been killed, the esophagus was ligated, stomachs excised, and gastric juice carefully collected in graduated tubes after removal of the esophageal ligature. The volume of gastric secretion was noted and gastric acid output determined by automatic titration to pH 7.0 with 0.01*N* NaOH and $H^+$ output expressed as μEq/4 h.

### Drugs and Chemicals

Pentagastrin (Peptavlon, ICI, U.K.), histamine dihydrochloride (BDH Chemicals, U.K.), indomethacin (Kahira Pharm & Chem. Ind. Co., Egypt), urethane (Sigma, U.S.), and alcian blue 8 GX (Sigma, U.S.) were used. Drugs were freshly dissolved with isotonic NaCl immediately before the experiments to obtain the necessary doses. Indomethacin was dissolved in 5% sodium bicarbonate solution.

### Statistical Analysis

Results were expressed as means ± SEM. Data were analyzed using one-way analysis of variance; $p$ values less than 0.05 were considered as significant.

## RESULTS

### Gastric Acid Secretion Studies

#### *Basal Gastric Acid Secretion*

Basal gastric acid secretion in unirradiated control rats was 278.3 ± 12.9 μEq/100 g/4 h after ligating the pylorus. Exposure to 3- or 6-Gy gamma irradiation led to significant increase in basal (nonstimulated) gastric acid secretion in the 4-h pylorus-ligated rat. The increase in $H^+$ output was most marked in those irradiated with 3 Gy of gamma irradiation and less after a 6-Gy dose (47.5 and 18.2%, respectively, compared with unirradiated controls) (Fig. 1). The volume of gastric secretion was also higher in 3- or 6-Gy irradiated rats compared with their unirradiated counterparts (5.2 ± 0.4, 6.7 ± 0.5 vs. 7.1 ± 0.7 ml 100 g/4 h).

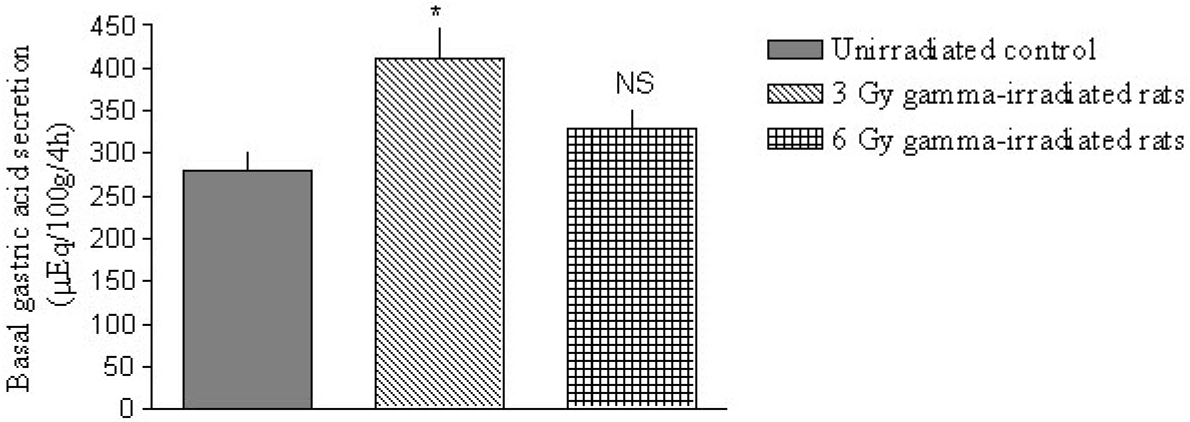

**FIGURE 1**. Effect of gamma irradiation on basal (nonstimulated) gastric acid secretion in the 4-h pylorus-ligated rat. Rats were subjected to 3- or 6-Gy gamma irradiation and 7 days later subjected to pylorus ligation together with i.g. 2 ml of physiological saline for 4 h. $^{*}p < 0.05$; NS: not significant vs. control (unirradiated group).

## Gastric Mucus

Gastric mucus indicated by alcian blue dye recovery was reduced by 8.2 and 16.7% in 3- or 6-Gy gamma irradiated rats compared to unirradiated rats (121.6 ± 8.6 and 110.3 ± 10.8 vs. 132.4 ± 10.6 μg/g).

## Pentagastrin-Stimulated Gastric Acid Secretion

Gastric acid secretion in the 4-h pylorus-ligated control (unirradiated) rats given pentagastrin (150 μg/kg) was 293.5 ± 22 μEq/4 h/100 g. On the other hand, in rats irradiated with 3- or 6-Gy fraction, gastric acid output on the 4-h pylorus-ligated rat following pentagastrin stimulation was markedly decreased by 67.4 and 72.8%, compared with unirradiated controls (Fig. 2).

## Histamine-Stimulated Gastric Acid Secretion

Under the condition of histamine (1 mg/kg) stimulation, gastric acid output in those irradiated with 3- or 6-Gy fraction of gamma rays was markedly lower (by 47 and 62.8%) than that in unirradiated rats (136.9 ± 13.3 and 115.7 ± 9.7 vs. 311.4 ± 19.6 μEq/100 g/4 h, respectively) (Fig. 3).

Histological examination of sections of rat gastric mucosa indicated superficial mucosal injury, although engorgement of microvessels with erythrocytes and some exfoliation of cells was seen in irradiated rats after stimulation with pentagastrin or histamine (Fig. 4).

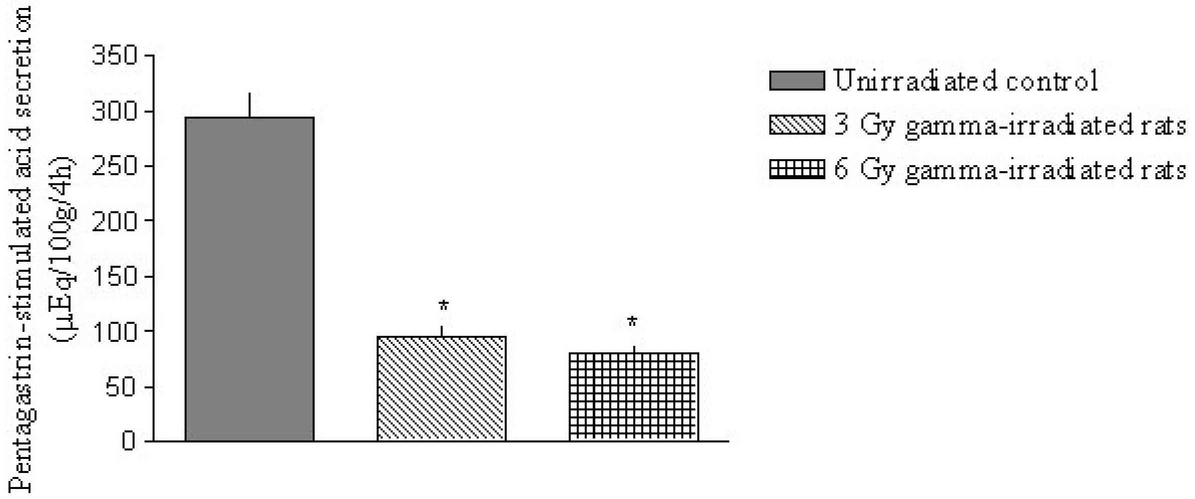

**FIGURE 2.** Effect of gamma irradiation on pentagastrin (150 μg/kg)-stimulated gastric acid secretion in the 4-h pylorus-ligated rat. Rats were subjected to 3- or 6-Gy gamma irradiation and 7 days later subjected to pylorus ligation and s.c. pentagastrin together with i.g. 2 ml of physiological saline for 4 h. *$p < 0.05$ vs. control (unirradiated group).

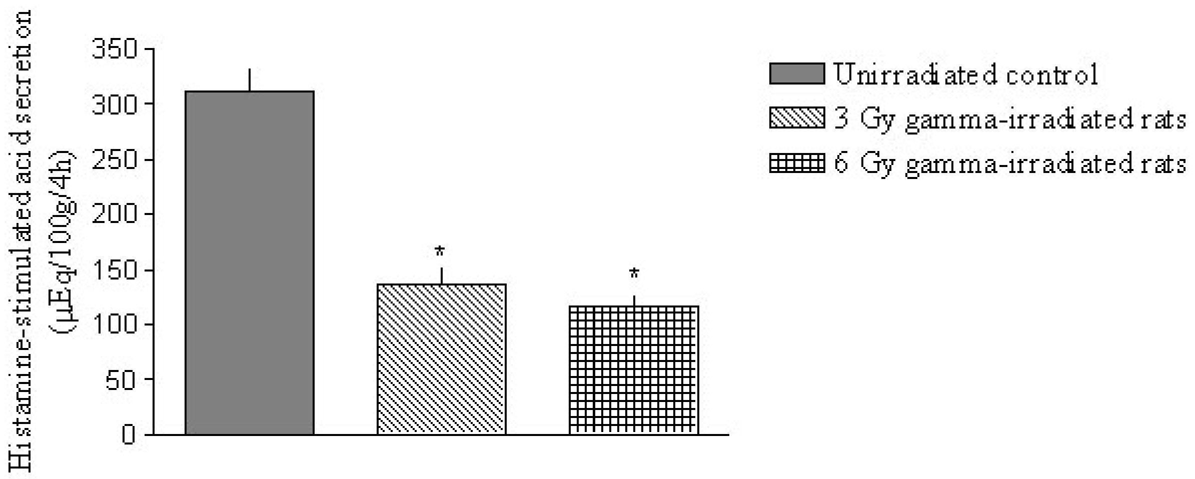

**FIGURE 3**. Effect of gamma irradiation on histamine (1 mg/kg)-stimulated gastric acid secretion in the 4-h pylorus-ligated rat. Rats were subjected to 3- or 6-Gy gamma irradiation and 7 days later subjected to pylorus ligation and s.c. histamine together with i.g. 2 ml of physiological saline for 4 h. *$p < 0.05$ vs. control (unirradiated group).

## Gastric Ulcer Studies

Both the number and severity of gastric mucosal lesions caused by indomethacin were markedly increased after 3- or 6-Gy irradition; the effect being dose dependent. Further, gastric acid output was reduced by 22 and 54.7% in rats irradiated with 3- or 6-Gy fraction (273.8 ± 26.7 and 159.2 ± 12.7 μEq/100 g/4 h) as compared with unirradiated controls (351.2 ± 19.6 μEq/100 g/4 h). The volume of gastric secretion was decreased in 3- or 6-Gy irradiated rats compared with their unirradiated counterparts (5.1 ± 0.4, 5.2 ± 0.5 vs. 6.4 ± 0.3 ml/100 g/4 h). Similarly, prior exposure to 3- or 6-Gy gamma irradiation exacerbated the degree of gastric mucosal injury caused by 50% ethanol in a dose-dependant manner (Table 1).

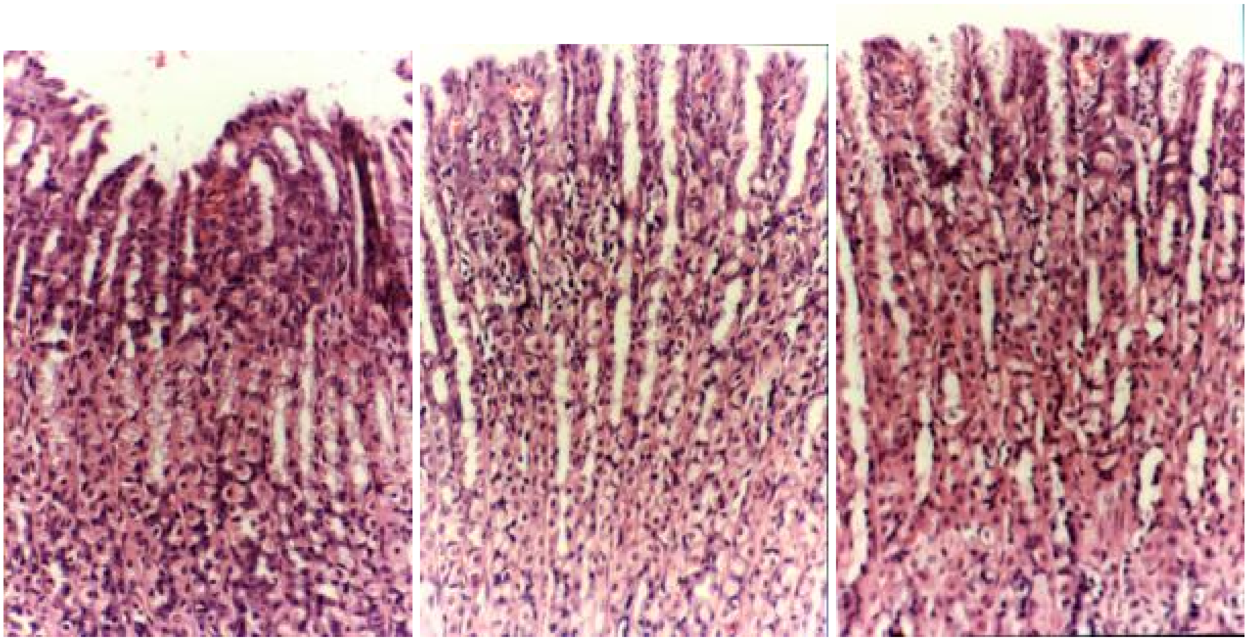

A B C

**FIGURE 4**. Photomicrograph of gastric mucosa from whole-body gamma-irradiated rats subjected to pylorus ligation and 2 ml of orogastric 0.9% saline for 4 h; (A) control saline, (B) pentagstrin treated, (C) histamine treated. The mucosa shows minimal superficial injury and engorgement of microvessels with erythrocytes; changes being most marked after pentagastrin or histamine stimulation (H & E stain).

**TABLE 1**
**Effect of a Single Dose (3 or 6 Gy) Whole-Body Gamma Irradiation on the Indomethacin- and Ethanol-Induced Gastric Mucosal Damage in 4-h Pylorus-Ligated Rats**

| Group | Number of Lesions/Rat/4 h | Severity of Lesions/Rat/4 h | Gastric Secretory Volume (ml/100 g/ 4 h) | Gastric Acid Output (μEq/100 g/4 h) |
|---|---|---|---|---|
| IND control | 1.8 ± 0.5 | 3.8 ± 1.2 | 6.4 ± 0.3 | 351.2 ± 26.7 |
| + 3 Gy | 2.4 ± 0.4* | 4.1 ± 0.7* | 5.1 ± 0.4* | 273.8 ± 23.3* |
| + 6 Gy | 4.7 ± 1.3$^{NS}$ | 7.8 ± 1.7* | 5.2 ± 0.3* | 159.2 ± 12.7* |
| Eth control | 4.0 ± 0.9 | 7.2 ± 1.7 | 2.5 ± 0.2 | 28.5 ± 3.9 |
| + 3 Gy | 7.7 ± 1.5* | 14.8 ± 2.2* | 2.9 ± 0.2$^{NS}$ | 35.7 ± 4.6$^{NS}$ |
| + 6 Gy | 12.8 ± 0.3* | 36.3 ± 3.4* | 2.9 ± 0.1$^{NS}$ | 32.3 ± 3.7$^{NS}$ |

Abbreviations: IND, indomethacin; Eth, ethanol. Statistical differences between control (unirradiated) and treatment (irradiated) groups were tested using one-way ANOVA and Duncan's multiple range test. A value of $p < 0.05$ was considered statistically significant. *$p < 0.05$; NS: not significant vs. control group.

## DISCUSSION

Data obtained in the present study from 3- or 6-Gy whole-body gamma-irradiated rats indicate that a significant increase in gastric acid secretion is registered in rats on the 7$^{th}$ day postirradiation. Under pentagastrin or histamine stimulation, however, gastric acid secretion in rats irradiated with 3-Gy fraction did not change, whereas those irradiated with 6-Gy fraction showed significantly lower acid output than unirradiated rats.

Studies indicated that exposure to irradiation could suppress gastric acid secretion. In man, decrease in gastric acid secretion was registered 10–14 days after irradiation of the lumbar area. Acid production indices came back to normal 3–3.5 months after irradiation[11]. Vaughan et al.[4] noted in anesthetized, acute gastric fistula rats a transient inhibition of spontaneous (nonstimulated) gastric acid secretion immediately after a dose of 240 R (2.4 Gy), whole-body X-ray irradiation, which resumed promptly after gastrin injection. In addition, gastrin-stimulated acid secretion was inhibited after a dose of 600 or 1200 R (6 or 12 Gy) X-ray irradiation. Man et al.[5] irradiated mice stomachs with 9-Gy X-rays and observed that under pentagastrin stimulation (62.5 μg/kg), acid and histamine secretion was significantly lower than that of the unirradiated controls 7 days after gastric irradiation. It was postulated that depletion of gastric histamine store contributes to the reduction in acid production, for in mice exposed to a single dose of 9 or 15 Gy of X-rays directed to the stomach, mean gastric mural histamine fell to 61 and 46% 7 days after irradiation[12]. In guinea pigs, total-body irradiation with 400 Gy suppressed gastric acid secretion under basal conditions and during histamine stimulation by 50–90%. Recovery from the radiation damage was only partial after 1 week[6]. In dogs, a decrease of $H^+$ production in the gastric juice was seen during the first 7 days after 12.7-Gy abdominal X-ray irradiation[13]. In rhesus monkeys, inhibition of gastric acid was evident for 2 h after total body exposure to 800-cGy $^{60}$Co but returned to preirradiation levels 2 days later. Irradiation produced an immediate significant increase in gastric juice concentration of PGE2 and PGI2 by 64.5 and 50.4%, respectively, which might have accounted for the radiation-induced suppression of acid output[14]. More recently, however, Lehy et al.[7] observed in awake gastric fistula rats an increase in basal acid secretion and plasma gastrin in rats at 3 and 7 days after exposure to 2- or 6-Gy single fraction gamma irradiation. Our data in pylorus-ligated rats are in agreement with those reported by Lehy and co-workers[7] and suggest that lower doses of irradiation are associated with increase in basal (nonstimulated) gastric acid secretion. The discrepancies in the literature regarding the effect of irradiation on gastric acid secretion are likely to arise from varying conditions, varying species, and different methods for assaying gastric acid output, and are much more likely to result from the variability in the doses of irradiation used. With higher doses of irradiation, the acute inflammatory response[1,2,15], impaired mucosal microcirculation[15], luminal release of prostaglandins[14], depletion of gastric histamine[5,12], as well as decrease of ATPase activity[13] are likely to account for the inhibition of gastric acid secretion observed in man and in acute animal experiments.

Although an increase in circulating gastrin by low doses of gamma irradiation[7] or sensitization to acetylcholine and histamine[16] might provide an explanation for the observed increase in acid output under basal conditions, the same mechanism cannot explain the opposite effect of irradiation on stimulated gastric acid secretion. The finding in the present study of a decrease in gastric acid output under pentagastrin or histamine stimulation and also after indomethacin in rats exposed to irradiation, could represent at least in part an increased permeability of an already-breached gastric mucosal barrier and enhanced $H^+$ back diffusion following the secretagogues or the ulcerogenic agent indomethacin.

The pylorus-ligated rat is a widely used model for the assay of gastric acid secretion, which permits the determination of gastric acid secretion within a defined period of time[17]. The hypersecretion of the pylorus-ligated rat is dependent on vagal activity being mediated by long vago-vagal reflexes for it can be abolished by vagotomy or atropine. Following the initial phase of hypersecretion of acid with the maximum volume and acid output reaching it maximum after 5–6 h, acid production ceases and actually diminishes 12–20 h after ligation[18,19,20], which is likely to be the result of depletion of energy stores and/or increased mucosal permeability. Vallgren and co-workers[21] suggested that both vago-vagal and intramural reflexes involve a cholinergic and histaminergic pathway that does not involve histamine derived from the gastric endocrine-like cells. Andersson et al.[22] further indicated that unlike pentagastrin stimulation, vagally stimulated gastric acid secretion induced by pylorus ligation was unaffected by depletion of histamine from the entrochromaffin-like cells [ECL] with alpha-fluoromethylhistidine. Ultrastructural studies by Zhao and co-workers[23] confirmed that ECL in the rat oxyntic mucosa do not mediate the gastric acid response to pylorus ligation, and that ECL cells in the pylorus-ligated stomach retain their ability to respond to gastrin with activation. Since histamine-forming capacity is markedly reduced in the stomachs of mice subjected to irradiation[12], it is thus possible that the present findings represent failure of the secretagogue (histamine or pentagastrin)-direct stimulation of parietal cell. This hypothesis assumes that direct stimulation of the parietal cell with histamine or pentagastrin overrides the reflexes caused by ligating the pylorus and that mucosal histamine plays a more important role in the action of gastrin than of cholinomimetics or pylorus ligation on the parietal cell, which has been suggested by different workers[21,22,24].

A likely explanation for the failure of the secretagogue (histamine or pentagastrin)-direct stimulation of parietal cell may be the decrease of adenosine triphosphatase (ATPase) activity[13] and the depletion of energy stores. The metabolic activity of the gastric mucosa is very high, owing mainly to the secretion of acid by parietal cells[25]. The secretion of HCl is an energy-demanding process, which is partly given by the transformation of adenosine triphosphate (ATP) into adenosine diphosphate (ADP) in the gastric mucosa, catalyzed by the membrane-bound ATPase. This transformation is facilitated by the effect of acetylcholine chloride on the transport ATPase system. This effect of acetylcholine chloride is prevented by atropine [26, 27, 28]. Irradiation alone can inhibit the metabolism of gastric cells and tissues. The activity of total ATP-ase and Mg-dependent ATP-ase in gastric mucosal cells is reduced shortly after irradiation [13, 29]. ATP plays a fundamental role in radioprotection and metabolic provision for repair processes [30]. Survival of the mice, using 30 days post-irradiation as the endpoint, was increased from 40% to 85% by action of the exogenous ATP [31, 32].

The absence of marked gastric mucosal injury in the stomachs of irradiated rats stimulated with histamine or pentagastrin compared with those treated with indomethacin may be due to increased blood flow in the former situation[33,34], which helps to neutralize the influxing acid load and supplies energy substrates and oxygen, thereby helping the gastric mucosa to resist the chemical injury. This crucial role of gastric mucosal blood flow in maintaining gastric mucosal integrity has been demonstrated in many studies[35,36,37,38,39].

In the case of damage to the gastric mucosa by ethanol, pentagastrin and histamine, while stimulating acid secretion, were able to reduce gastric mucosal damage due to ethanol[26]. Increase in metabolic activity by pentagastrin and histamine help to prevent the chemical-induced injury to the gastric mucosa[26]. It has been shown that the actively secreting gastric mucosa has an increased resistance to noxious chemical injury[26,40]. Conversely, after irradiation and due to depletion of energy stores, the mucosal damage due to chemical injury is likely to be exacerbated, as can be seen in the present study, where damage due to ethanol or indomethacin is intensified in rats subjected to irradiation. Changes in gastric mucus can alter the susceptibility of the gastric mucosa to injury[41]. A reduction in gastric mucus by irradiation shown in the present work may be an additional mechanism by which irradiation enhanced the indomethacin and ethanol damage to gastric mucosa.
